# An unusual cause of postmenopausal vaginal haemorrhage: a case report

**DOI:** 10.1186/s12905-019-0731-4

**Published:** 2019-02-07

**Authors:** Junyan Sun, Ying Guo, Li Ma, Zhaoxia Qian, Dongmei Lai

**Affiliations:** 1 The International Peace Maternity and Child Health Hospital, School of Medicine, Shanghai Jiaotong University, 145, Guang-yuan Road, Xuhui District, Shanghai, 200030 China; 2 Zhangshan Hospital, Fudan University, 180, Fenglin Road, Xuhui District, Shanghai, 200032 China

**Keywords:** Postmenopausal vaginal bleeding, Vaginal varicosities, Deep arteriovenous shunts

## Abstract

**Background:**

Post-menopause vaginal haemorrhage is typically related to gynaecological malignancies. Bleeding from vaginal varices rarely occurs, especially in nonpregnant women. Moreover, nonpregnancy-related causes of vaginal varicosities include portal hypertension, especially that caused by liver cirrhosis, pelvic congestion syndrome and Klippel-Trenaunay syndrome or Parkes-Weber syndrome. Here, we report an unusual cause of nonpregnancy-associated vaginal variceal bleeding.

**Case presentation:**

A 55-year-old postmenopausal woman presented in our outpatient department with complaints of recurrent bloody vaginal discharge. A group of varicose veins and several haemorrhagic spots were found on her vaginal wall during a vaginal speculum examination. Genital cancers were excluded by colposcopy and transvaginal ultrasonography; furthermore, a pelvic arteriovenous fistula was not found on a pelvic computed tomography (CT) scan. However, congenital varicosities and deep arteriovenous shunts were observed in her left leg on arterial angiography. Moreover, vaginal bleeding was improved after resolution of the underlying deep arteriovenous shunts in her left leg. Therefore, congenital arteriovenous shunts and elevated inferior vena cava pressure might be responsible for her recurrent vaginal varicose bleeding.

**Conclusion:**

Haemorrhage due to vaginal varices is easily detected with a vaginal speculum examination. However, diagnosis and treatment of the original disease are important after bleeding is controlled.

## Introduction

Postmenopausal vaginal bleeding is a frequent medical problem with a prevalence rate as high as 10% in the general population [1, 2]. The causes of postmenopausal bleeding include vaginal or endometrial atrophy, hormone replacement therapy (HRT), endometrial cancer, endometrial or cervical polyps, and endometrial hyperplasia [3, 4]. Vaginal varicosities are part of a larger set of complications that can occur as a result of venous congestion and obstruction in both pregnant and nonpregnant patients. The occurrence of vaginal varicosities during pregnancy is less common than the occurrence vulvar varicosities, which occur in 2–4% of pregnancies [5–7]. Nonpregnancy-related causes of vaginal varicosities include portal hypertension, especially that caused by liver cirrhosis, pelvic congestion syndrome and Klippel-Trenaunay syndrome or Parkes-Weber syndrome [8–11]. Here, we report a postmenopausal woman with vaginal varicose bleeding who presented to our outpatient department and had neither abnormal liver function nor pelvic congestion syndrome.

## Case presentation

**Fig. 1:**
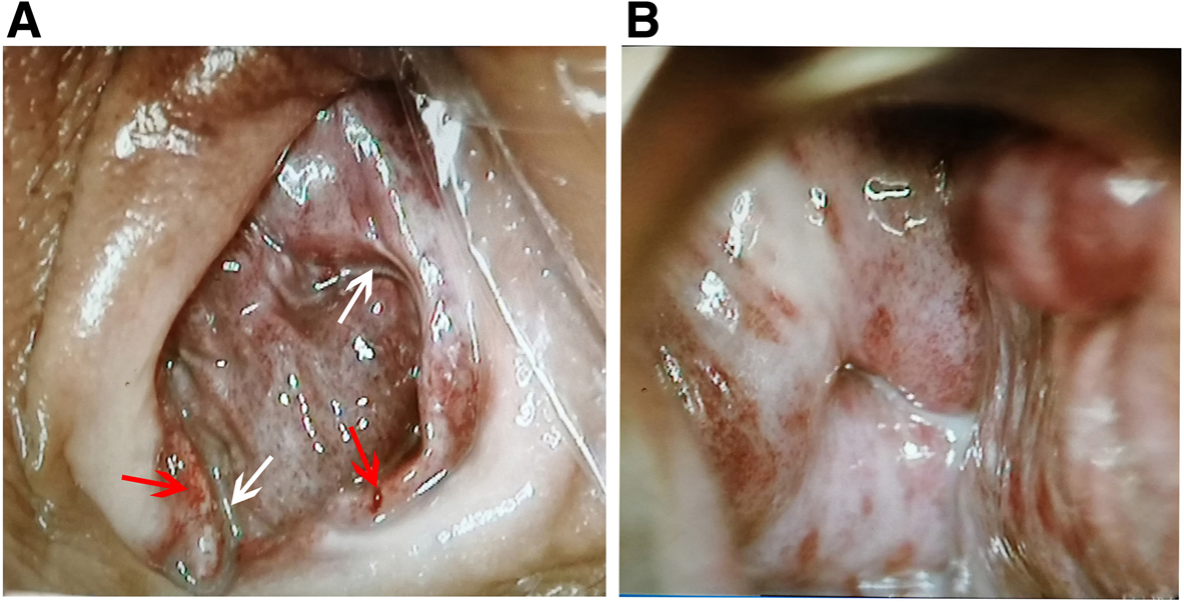
Iodine staining of the vaginal wall and cervix as shown by colposcopy. **a** The vaginal wall exhibited many varicosities and some bleeding spots. **b** Uterine cervix with iodine staining; no lesions were found in the cervix. White arrows: varicosities in the vaginal wall. Red arrows: bleeding spots

A 55-year-old postmenopausal woman presented in our outpatient department because of increasingly serious vaginal bleeding in the previous 2 years. She had experienced a caesarean delivery at 28 years old and underwent natural menopause at 53 years old. The bleeding, which followed a severe cough, was less than normal menses at first but later was heavy and left her clothes soaking wet. The patient reported that the bleeding was fresh blood, which could be stanched by compression in a sitting position. At the time of admission, a vaginal speculum examination showed that the vaginal wall had many varicosities and some bleeding spots. To exclude malignant diseases, the patient underwent colposcopy, and the varicosities and bleeding spots were further confirmed in the vaginal mucosa (Fig. 1a). However, iodine staining showed no lesions on the mucosae (Fig. 1b). In addition, the endometrial thickness was less than 4 mm according to transvaginal ultrasonography.

**Fig. 2:**
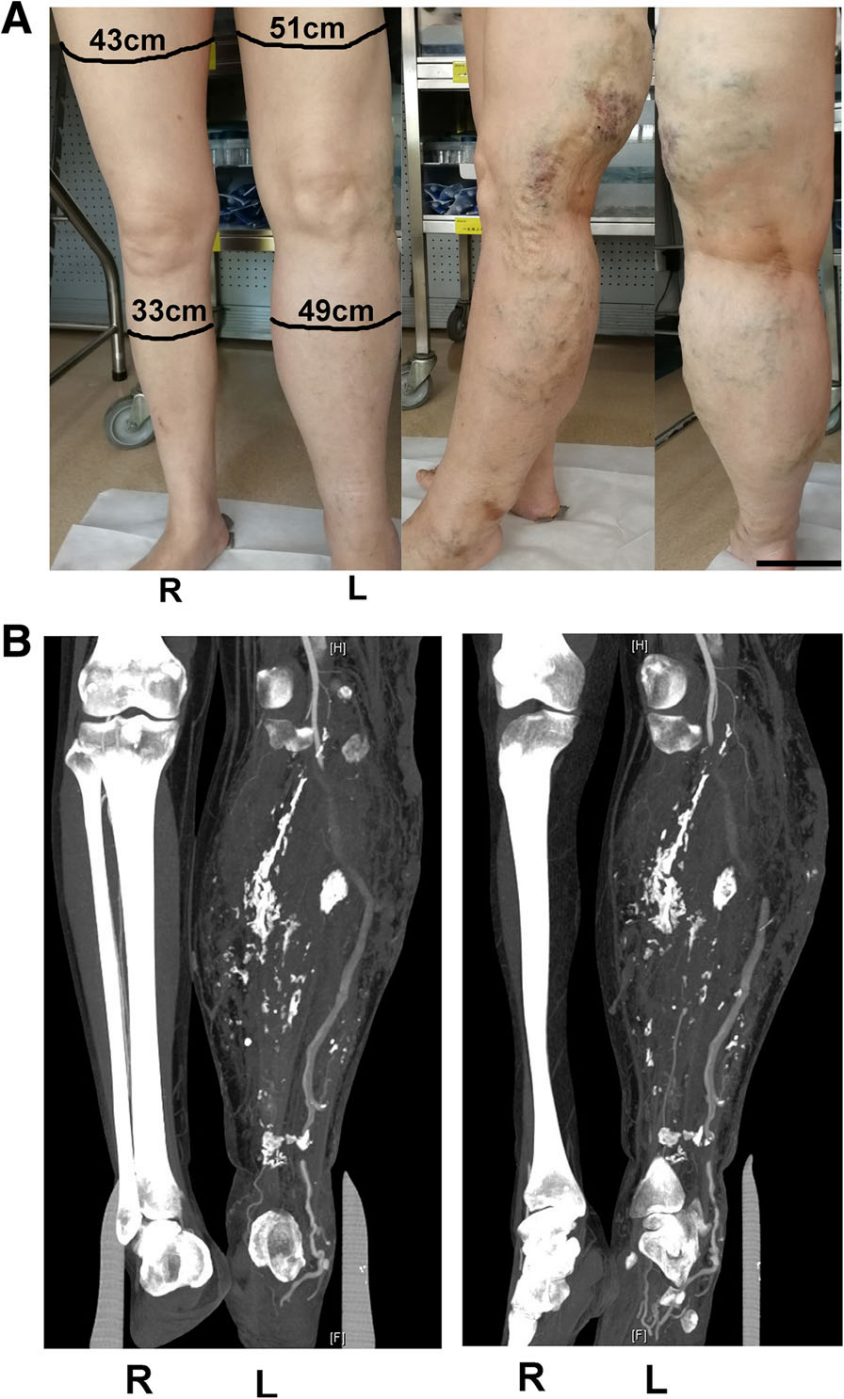
Lesions in the left lower limb. **a** General morphology of the legs of the patient. Front position: the right leg was larger than the left leg, and the circumference is shown in the figure. Lateral position: numerous varicosities and rough skin on the left leg. **b** Contrast-enhanced computed tomography (CT) scan of the lower limbs: front and lateral positions. R: right leg; L: left leg

**Fig. 3:**
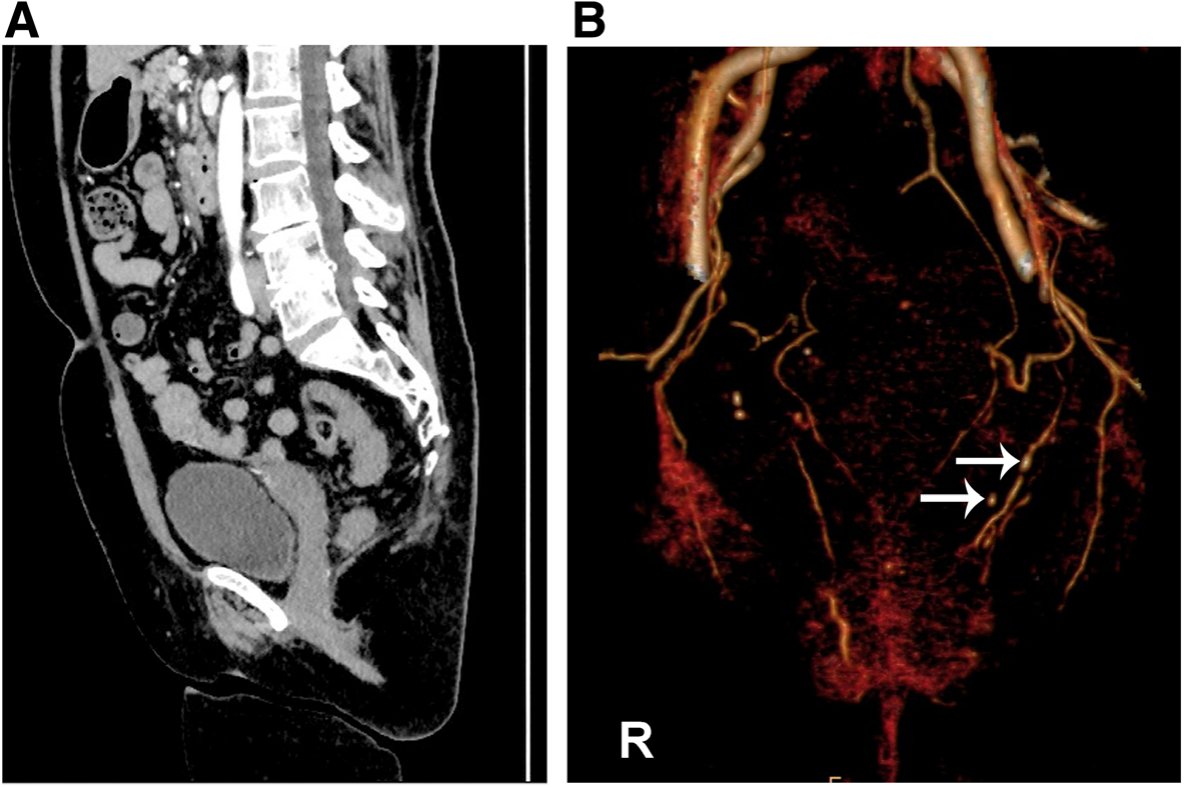
Lesions in the pelvic cavity. **a** Contrast-enhanced computed tomography (CT) scan of the abdomen and pelvic cavity. **b** 3-D reconstruction of the pelvic vessels. White arrows: enlarged vein and varicosities in the left pelvic region. R: right side

Simultaneously, we noticed that the patient had obvious venous varicosities in her left leg and that the diameter of the left leg was significantly larger than that of the right leg (Fig. 2a). She stated that the venous varicosities were found when she was 10 years old but not treated because of poor medical conditions. To exclude vascular malformation, we advised the patient to visit the vascular surgery department. On contrast-enhanced computed tomography (CT) scanning of the legs, several varicosities and hyperplastic soft tissue and muscle were found in the left leg but not in the right leg (Fig. 2b). To determine whether multiple arteriovenous malformations were present, the patient underwent a CT scan of the pelvis, which showed some venous varicosities in the left internal pudendal vein but not in the right vein (Fig. 3a). However, pelvic arteriovenous shunts and pelvic congestion syndrome were not found on the 3-D reconstructed image (Fig. 3b).

Then, deep arteriovenous shunts in the left leg were found by arterial angiography. The arteriovenous shunts were partly closed by an interventional arteriovenous fistula occlusion operation at Zhongshan Hospital, Fudan University, Shanghai, China. Aspirin and Plavix were given after surgery as anticoagulants for 1 month and were gradually stopped over the next 2 months. The vaginal bleeding was gradually alleviated and disappeared 5 months later. For long-term management, close observation and regular follow-up examinations were recommended.

## Discussion and conclusion

Postmenopausal bleeding is often associated with either a benign or malignant endometrial abnormalities [12]. However, haemorrhages from vaginal varices have been reportedly caused by arteriovenous malformations secondary to portal hypertension in cirrhotic patients and pelvic congestion syndrome in postmenopausal women [8, 11, 13]. Bleeding caused by increased central venous pressure, as in this case, has rarely been reported. However, we could not exclude the possibility of Parkes-Weber syndrome in this patient, which is a clinical disorder consisting of capillary malformation, soft tissue and bone hypertrophy, venous and lymphatic malformations and/or mutations in the RASA 1 gene [14, 15]. Although the patient had vaginal varices, congenital arteriovenous malformations and soft tissue hypertrophy in the left limb, there was no evidence of capillary malformation or RASA 1 gene mutation.

Vulvovaginal varices are often found during pregnancy, usually developing after 12–26 weeks of pregnancy, and largely self-resolve shortly after delivery [16–18]. Pregnancy itself causes several physiological changes that favour varicosity formation. Much of the literature focuses on vulvar varicosities during pregnancy because vaginal venous flow occurs via a venous plexus that communicates with numerous plexuses, such as the vesical and haemorrhoidal plexuses [19]. Furthermore, vulvovaginal varicosities are aggravated by delivery, as described by Pratilas and colleagues [20]. In that case, the 34-year-old patient carried one male foetus delivered via C-section, and the vaginal varicosities were found during pregnancy. However, the symptoms did not spontaneously resolve during the postpartum period.

In most cases, the treatment of vaginal variceal bleeding comprises two stages: controlling the haemorrhage and, subsequently, determining the cause and curing the underlying disease [8, 21]. In this case, when the patient initially visited our department, the vaginal bleeding had stopped. At that time, benign or malignant genital diseases were excluded. Considering her recurrent vaginal bleeding, we advised the patient to seek treatment for the underlying disease. Five months after her limb surgery, the bleeding was significantly reduced. Thus, the vaginal bleeding might have occurred secondary to the increased central venous pressure caused by the arteriovenous shunts in her leg.

Vaginal variceal bleeding decreases the quality of life, especially in postmenopausal women. First, vaginal bleeding should be considered when the discharge consists of fresh blood. Then, a vaginal speculum examination should be carefully conducted. Furthermore, it is important to determine and cure the underlying disease after the bleeding is controlled.

## Abbreviations

CT: Computed tomography
HRT: Hormone replacement therapy
